# Supplementary material for: Plasma-Activated Polydimethylsiloxane Microstructured Pattern with Collagen for Improved Myoblast Cell Guidance
Source: Int J Mol Sci. 2024 Feb 28;25(5):2779. doi: 10.3390/ijms25052779 (PMC10932060; doi:10.3390/ijms25052779)
Supplement: Supplementary file 1 [file ijms-25-02779-s001.zip › ijms-2869607-supplementary.pdf]

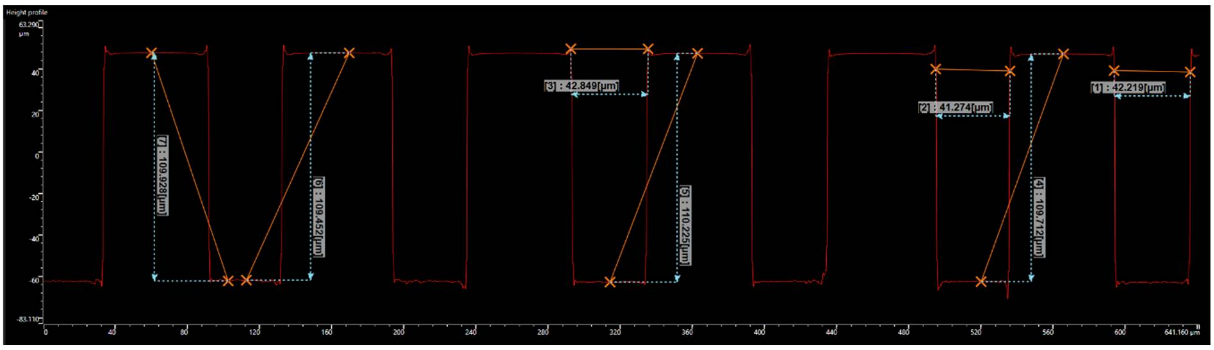

Figure S1: Detail of height profile measurement of photoresist patterns using software iQ-Analyzer-X

(a)

(b)

(c)

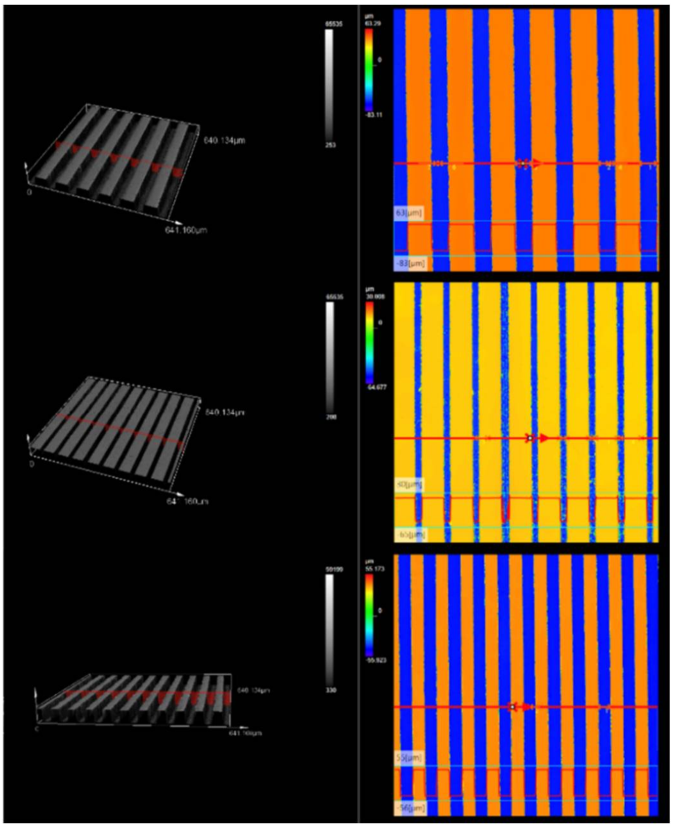

Pattern dimension	Space dimension	Height
50 x 50 µm	43 µm	110 µm
50 x 20 µm	17 µm	63 µm
30 x 30 µm	31 µm	83 µm

Figure S2: Visual representation of height profile measurements of photoresist patterns from iQ-Analyzer-X software. (a) Photoresist microstructure with a pattern size of 50 x 50 µm, (b) 50 x 20 µm, and (c) 30 x 30 µm.

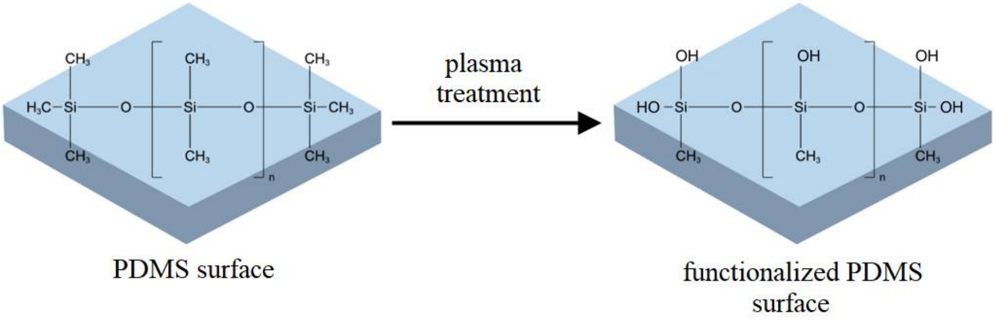

Figure S3: Effect of plasma modification on PDMS surface [98].

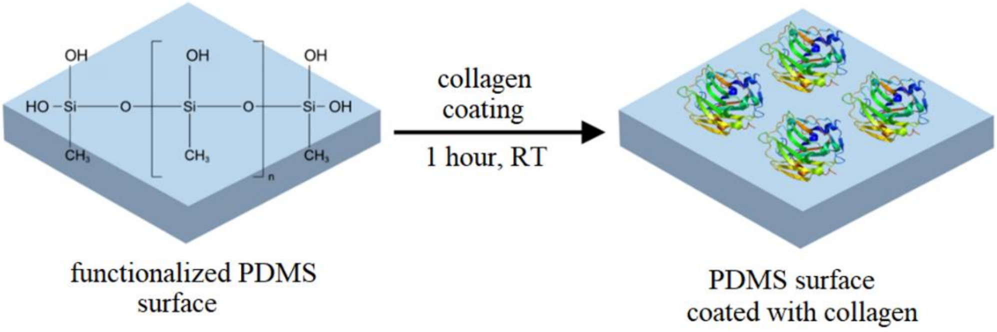

Figure S4: Coating of the functionalized PDMS surface with type I collagen. The incubation time of the coating was 1 h at room temperature
